# Assessment of the Genetic Potential of the Peregrine Falcon (*Falco peregrinus peregrinus*) Population Used in the Reintroduction Program in Poland

**DOI:** 10.3390/genes12050666

**Published:** 2021-04-29

**Authors:** Karol O. Puchała, Zuzanna Nowak-Życzyńska, Sławomir Sielicki, Wanda Olech

**Affiliations:** 1 Department of Animal Genetics and Conservation, Warsaw University of Life Sciences, 02-787 Warszawa, Poland; zuzanna_nowak_zyczynska@sggw.edu.pl (Z.N.-Ż.); wanda_olech@sggw.edu.pl (W.O.); 2 Society for Wild Animals “Falcon”, 87-800 Włocławek, Poland; falco@peregrinus.pl

**Keywords:** microsatellites, populational genetics, *Falco peregrinus*

## Abstract

Microsatellite DNA analysis is a powerful tool for assessing population genetics. The main aim of this study was to assess the genetic potential of the peregrine falcon population covered by the restitution program. We characterized individuals from breeders that set their birds for release into the wild and birds that have been reintroduced in previous years. This was done using a well-known microsatellite panel designed for the peregrine falcon containing 10 markers. We calculated the genetic distance between individuals and populations using the UPGMA (unweighted pair group method with arithmetic mean) method and then performed a Principal Coordinates Analysis (PCoA) and constructed phylogenetic trees, to visualize the results. In addition, we used the Bayesian clustering method, assuming 1–15 hypothetical populations, to find the model that best fit the data. Units were segregated into groups regardless of the country of origin, and the number of alleles and observed heterozygosity were different in different breeding groups. The wild and captive populations were grouped independent of the original population.

## 1. Introduction

The peregrine falcon can be found on all continents except Antarctica. A decrease in the *Falco peregrinus* population in Europe occurred mainly in the 1950s and 1960s [1]. Wide use of DDT (dichlorodiphenyltrichloroethane) and other persistent chlorinated insecticides in agriculture led to the bioaccumulation of these chemicals. The accumulation of DDT and other persistent chlorinated insecticides resulted in ever thinner falcon eggshells [2], leading the peregrine falcon to the brink of extinction. In Europe, the only subspecies not to suffer from a significant reduction in its population size was *F. p. pealei*. It is assumed that this was due to the different diet of this cliff-dwelling subspecies [3,4,5].

Despite the peregrine falcon’s (*Falco p. peregrinus)* presence all over Poland, the population size has never been very large [6]. Following the population decrease in the 1950s, the last known nests of the species were observed in 1964 [7]. Between 1970 and 1985, only one breeding pair was recorded; this was in 1980 in the Tatra Mountains [8]. Poland’s peregrine falcon population has mostly been of the tree-nesting ecotype (also called the “forest ecotype”). The range of the population of tree-nesting ecotype peregrine falcons is from northeast Germany through Poland and Belarus to central Russia (central and eastern Europe), and this ecotype became virtually extinct in the 1950s. Between 1950 and 2007, only one pair of tree-nesting peregrine falcons was reported [9,10]. Likewise, in Germany, there were no confirmed sightings of tree-nesting peregrine falcons until the mid-1990s [11]. The tree-nesting ecotype peregrine falcon belongs to the same subspecies (*Falco p. peregrinus*) as the urban ecotype. The ecotype of a falcon is determined by the nesting site: falcons of the tree-nesting ecotype nest in trees, while birds of the urban ecotype prefer sites similar to cliffs or mountainside-like tops of skyscrapers or industrial chimneys. Despite the fact that the conditions conducive to tree nesting occur in places other than the above-mentioned fragment of Europe, there is no evidence of tree nesting in other areas. Furthermore, there is no evidence of phylogenetic differences between ecotypes [12,13]. Recent studies have shown that a mechanism of “imprinting” on the place of birth occurs, whereby birds that have hatched in tree nests, after leaving their parents’ nest, make nests in areas similar to those in which they were born [7].

The development of methods for breeding falcons in captivity in the 1960s made reintroduction programs possible [7]. In some countries of continental Europe and in the UK, reintroduction programs began in the 1970s with successful programs restoring the peregrine falcon population in some countries to pre-crisis numbers [2,14,15] The breeding of peregrine falcons in Poland began around the late 1980s with the revival of Polish falconry. The birds in the newly established Polish breeding centers originated from West European breeding sources and represented the *Falco p. peregrinus* subspecies [7,12]. The reintroduction of peregrine falcons in Poland began in 1990, and in 1992, cooperation between breeding centers and institutions participating in their restoration commenced under the title “Program for the restitution of the peregrine falcon (*Falco peregrinus peregrinus*) population in Poland.” [7]. The aim of this program was to establish a stable and functioning population of peregrine falcons across Poland. In the years 1990–1994, 51 young falcons were released [16]. By 2009, 345 peregrine falcons had been released into the wild, and in 2010, the program was taken over by the Society for Wild Animals “Falcon”, which is a non-governmental organization that is still responsible for peregrine falcon restitution in Poland today. Since 2010, a total of 879 individuals have been released into the wild, and the number of falcons bred in Poland has increased constantly [13].

Some peregrine falcon populations have been subjected to genetic variance analysis. Using samples from a Scandinavian population, Nesje et al. [17] described the genetic markers used for genetic variance analyses of peregrine falcons and other Falconidae members [17,18,19,20]. Nittinger et al. [18] used this panel of markers in addition to mitochondrial genotyping to describe the genetic structure of the saker falcon (*Falco cherrug*) and the low genetic variance among this species. In Scandinavia, low genetic variance was found in a contemporary population as well as in a historical population from museal collections [19]. Bryndova et al. [1], analyzed wild and captive populations of peregrine and saker falcons living in the Czech Republic, and saker falcon significant differentiation between captive and wild birds was found. However, no significant differences were observed between peregrine falcons. Some peregrine falcon populations have been subjected to genetic variance analysis. The process of “gene flow” in dispersed populations shows that no continental subspecies is genetically isolated from another [20], but an analysis of two rural and two urban groups in a Polish population of European kestrels showed significant genetic differentiation between the analyzed groups [21].

## 2. Materials and Methods

### 2.1. Sampling and DNA Extraction

**Figure 1:**
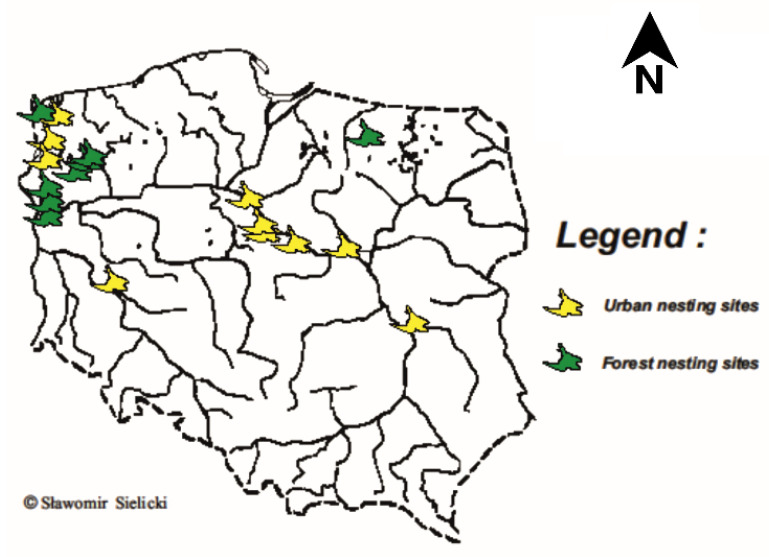
Map showing sampling locations from wild birds.

Peripheral blood was collected from wing veins by needle puncture, and this was inserted into test tubes containing 96% ethanol (Approval numbers 3181/2015 and 3445/2015). Blood samples and feathers were collected from 353 peregrine falcons, both captive (*n* = 262) and wild birds (*n* = 91) of the wild bird tree-nesting ecotype (*n* = 34) and urban nesting (*n* = 57) ecotype. Birds were assigned to ecotypes on the basis of observation of the place of nesting. The wild individuals originated from the Polish population and were collected from 19 nesting sites, 9 forest nests and 10 urban nests, as shown in Figure 1. The samples from captive birds were obtained from 47 breeders from 5 different countries—Poland (*n* = 86), Czech Republic (*n* = 91), Germany (*n* = 54), Slovakia (*n* = 21), and Denmark (*n* = 10)—prior to the birds being released into the wild as part of a restitution program. For genetic variation analyses, individuals were divided into 6 groups, 5 containing captive birds divided by country of breeding and 1 containing wild birds.

DNA was extracted from blood and feathers using the NucleoSpin Tissue mini kit (Macherey-Nagel, Düren, Germany) in accordance with the manufacturer’s instructions. For DNA extraction from feathers, the duration of incubation with proteinase was extended to 12 h.

### 2.2. Microsatellite Genotyping

Microsatellite genotyping was performed for all individuals. Microsatellite markers were amplified with primers for *F. peregrinus* [1,17,18,19,22] (GenBank sequence accession numbers AF118420-AF118434 [17]). Based on the related body of literature, markers meeting the following criteria were selected for analysis: the possibility of performing the analysis through one Multiplex PCR reaction and the presence of a high locus polymorphism. The primer melting point and product length range were checked for all markers. The following markers were amplified with fluorescent primers that were labelled with 6-FAM, PET, VIC or NED: NVHfp5 (labelled with VIC), NVHfp13 (labelled with 6-FAM), NVHfp46_1 (labelled with VIC), NVHfp54 (labelled with PET), NVHfp79_4 (labelled with 6-FAM), NVHfp82_2 (labelled with VIC), NVHfp86_2 (labelled with PET), NVHfp89 (labelled with6-FAM), NVHfp92 (labelled with NED), and NVMfp107 (labelled with NED). Multiplex PCR was performed in 7 µL volume reactions, each reaction containing the following: 1 µL of DNA (50–100 ng), 3.5 µL of Master Mix, 0.35 µL of Q-solution (Qiagen^®^), 0.93 µL of H_2_O, and primers in the following volumes (which were equal for reverse and forward primers and concentration of 100 pm/µL): NVHfp5, 0.056 µL; NVHfp13, 0.056 µL; NVHfp46_1, 0.05 µL; NVHfp54, 0.070 µL; NVHfp79_4, 0.056 µL; NVHfp82_2, 0.042 µL; NVHfp86_2, 0.105 µL; NVHfp89, 0.056 µL; NVHfp92, 0.056 µL; NVHfp107, 0.042 µL. The reaction was performed in a Biometra T3 thermocycler. The cycling conditions were as follows: 14.5 min at 95 °C; followed by 15 cycles at 95 °C for 30 s, 58 °C decreasing every cycle by 0.2 °C for 30 s, 72 °C for 1 min and then 20 cycles at 95 °C for 30 s, 55 °C for 30 s, 72 °C for 1 min, and then 60 °C for 30 min. The samples were then kept 4 °C until collection.

PCR products were mixed with size standard and formamide loading buffer. An ABI3500 DNA analyzer was used to visualize the PCR products. Allele sizes were assigned using GENEMAPPER 4.0 software produced by Applied Biosystems Inc. GENEMAPPER 4.0 is a fragment analysis software for Applied Biosystems^®^ genetic analyzers, which analyzes the quality of obtained fragments and assigns the analyzed fragments to specific loci based on the length of the fragments and the type of dye. Lengths of obtained fragments represent specific alleles.

### 2.3. Statistical Analyses

Genotypes were tested for departure from the Hardy–Weinberg equilibrium using GenAlEx v6.5 software (Genetic Analysis in Excel) [23,24] by performing a Chi-Square Test of the Hardy–Weinberg Equilibrium. Testing was performed for samples from 5 countries in which independent breeding occurs and for all pooled samples.

The basic analysis, featuring genotype comparisons, heterozygosity calculation, and the estimation of allele numbers, was performed using GenAlEx and Excel 2016 v16.0.12901.20462 (Microsoft) software. The probability of the occurrence of null alleles was estimated using Cervus 3.0.7 [25].

The genetic distance among populations was calculated using Nei’s [26] genetic distance method, which was performed with GenAlEx. A Principal Coordinates Analysis (PCoA) based on Nei’s genetic distance matrix was performed for data visualization in GenAlEx. The F-statistics for all analyzed samples and pairwise F-statistics were calculated with GenAlExPhylogenetic trees based on Nei’s genetic distance matrix, which was constructed using the UPGMA [27] and the Neighbor-Joining method [28] using Mega X v10.2.4 [29].

In STRUCTURE v2.3.4 [30,31,32,33], the Bayesian clustering method was used. This method assumes the Hardy–Weinberg equilibrium (HWE) is present in the population and uses allele frequencies from multilocus genotype data and Markov chain Monte Carlo (MCMC) sampling to assign individuals to a given number of clusters (K) [32]. Analyses were performed for K in the range 1–15 in 3 repetitions for each value of K. The Length of Burn-in Period was set to 50,000, and the Number of MCMC Reps after Burn-in was set to 500,000. Next, the results from STRUCTURE v2.3.4 were analyzed in the STRUCTURE Harvester v0.6.94 [34] program, which processes the results from STRUCTURE and executes the “Evanno” method [35]. Evanno plots from the STRUCTURE Harvester enable one to detect the number of K groups that best fits the data [34].

## 3. Result

Multilocus genotypes were obtained in 10 loci. A total of 326 out of 353 individuals were genotyped in all loci, 23 in 9 loci and 4 in 8 loci. No null alleles were found. Every individual had a unique multilocus genotype. The number of alleles per locus in the analyzed groups ranged from 2 to 9. The mean heterozygosity observed for each country of breeding and the wild population ranged from 28.9% to 41.2%, with an average value of 35.1%. Private alleles were observed in five samples across four populations in five different loci. The marker NVHfp5 was found to be monomorphic in the Danish population.

Genetic variability factors across all samples are presented in Table 1. The pairwise F_ST_ varied from 0.008 (for the Poland captive-Czech Republic pair) to 0.081 (for the Poland wild-Denmark pair) with an average value of 0.035. The highest F_ST_ values were obtained for pairs containing Denmark (mean value 0.063). For each of these pairs, the calculated F_ST_ was greater than for any other pair (the mean when pairs containing Denmark were excluded was equal to 0.021). This may have been caused by the low number of individuals from Denmark included (*n* = 10). In addition, one of these individuals was properly genotyped in nine loci, while the genotype with the NVHfp92_1 locus was not obtained.

**Figure 2:**
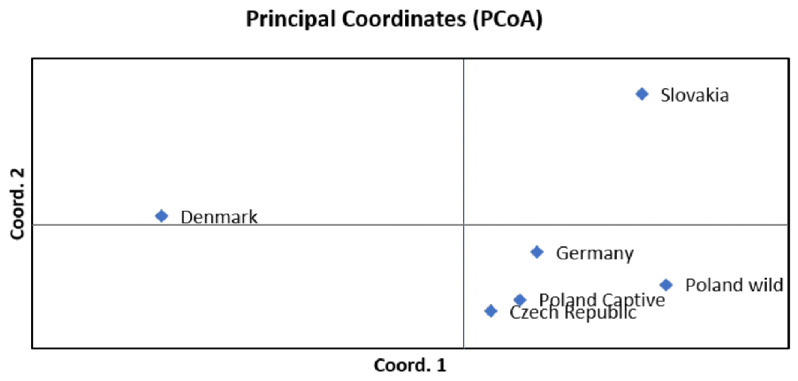
Principal Coordinates Analysis (PCoA) plot showing the genetic distance between groups.

Nei’s genetic distance varied from 0.018 (for the Poland captive-Czech Republic pair) to 0.207 (for the Poland wild-Denmark pair) with an average value of 0.082. Similar to F_ST_, highest Nei’s genetic distance values were obtained for pairs containing Denmark (mean value 0.152). Figure 2 shows the results of the PCoA analysis of Nei’s genetic distance. In the chart, axis 1 explains 87.32% of the variation and axis 2 explains 7.42% of the variation present. The cumulative percentage of variation explained by Figure 2 is equal to 94.74%.

**Figure 3:**
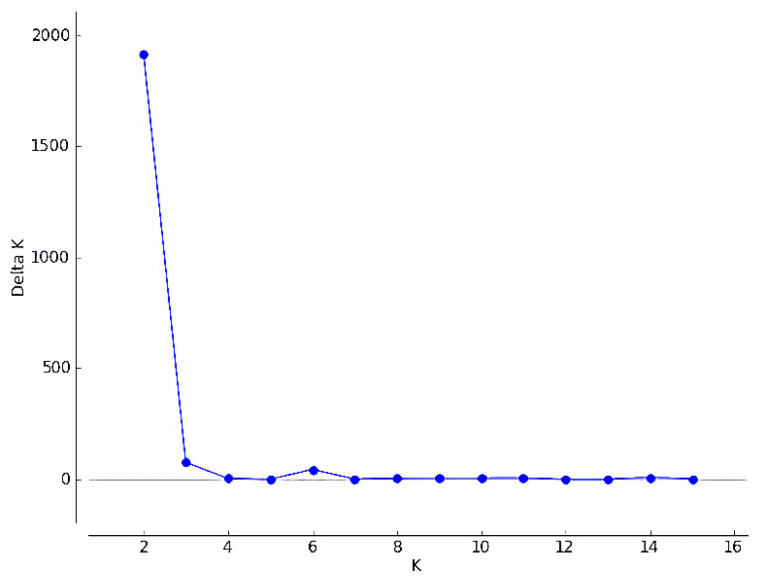
Delta K plot showing the value of K (number of groups within a population) that best fits the genetic data.

**Figure 4:**
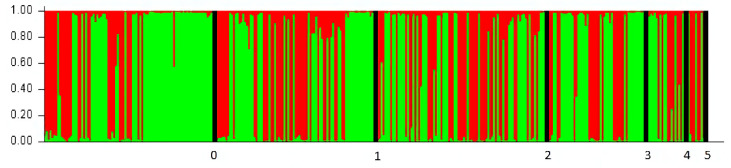
“STRUCTURE” plot that shows the genetic affiliation to groups computed by the program. Each column represents one individual and each color represents percentage of affiliation of individual to one genetic group estimated by program, green—group 1, red—group 2. Individuals are ordered by origin: 0—Poland wild, 1—Poland captive, 2—Czech Republic, 3—Germany, 4—Slovakia, 5—Denmark.

The best fit for the data determined by analyzing the STRUCTURE results with the “Evanno” method obtained for K = 2 groups (Figure 3). As Figure 4 shows, there were individuals belonging to both groups estimated by the program. It seems that the country of breeding did not affect group assignment. One bird from the Czech Republic was not assigned to a group due to its genetic similarity to both groups (~50% for both groups).

**Figure 5:**
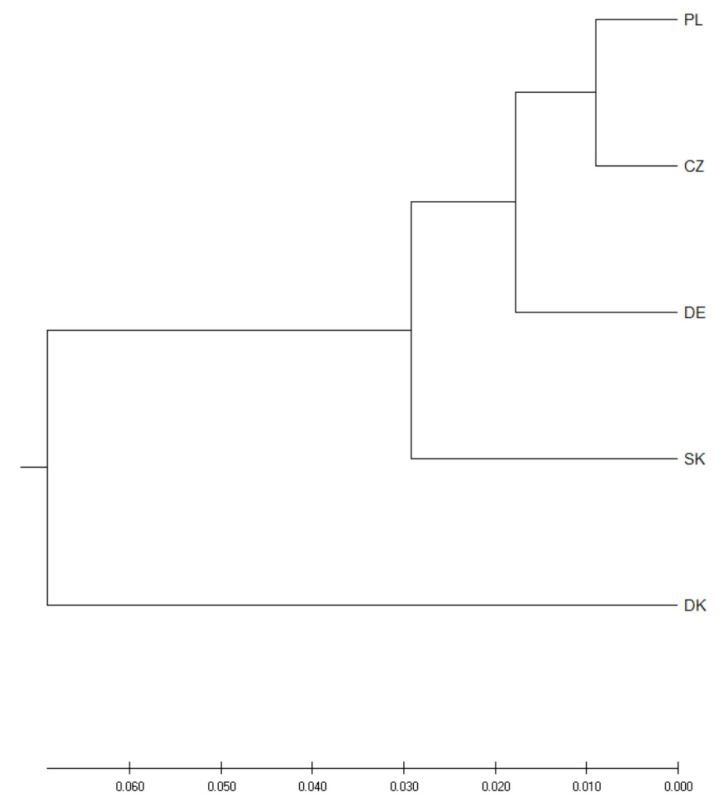
Nei’s distance relationships among breeding groups. The distances were inferred using the UPGMA method. The optimal tree with a sum of branch length of 0.19396867 is shown. The tree is drawn to scale with branch lengths having the same units as those of the Nei’s distances used to infer the distance matrix. PL—Poland, CZ—Czech Republic, DE—Germany, SK—Slovakia, DK—Denmark.

The genetic distance tree (Figure 5) containing birds from different breeding places in Europe shows trends in the exchange of birds between breeders and is consistent with the pedigree documentation.

**Figure 6:**
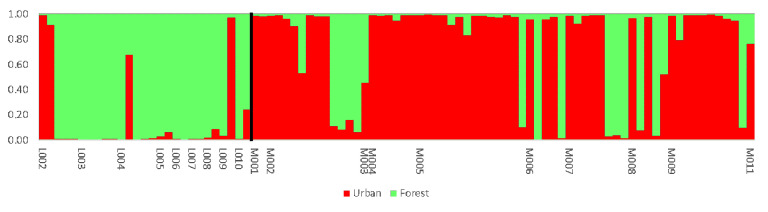
“STRUCTURE” plot that shows genetic affiliation to groups computed by the program. Each column represents one individual and each color represents one genetic group. Individuals were ordered by ecotype and nest: U—Urban nest, L—Forest nest, the numbers shown the different nests.

When only wild birds divided by ecotype (urban or tree-nesting) were analyzed, according to the STRUCTURE Harvester, the variability in the population was best described by division into two groups (delta K = 383.190615). Forty out of 57 urban ecotype individuals were classified into group 1, while 23 out of 34 tree-nesting individuals were classified into group 2 (Figure 6).

**Figure 7:**
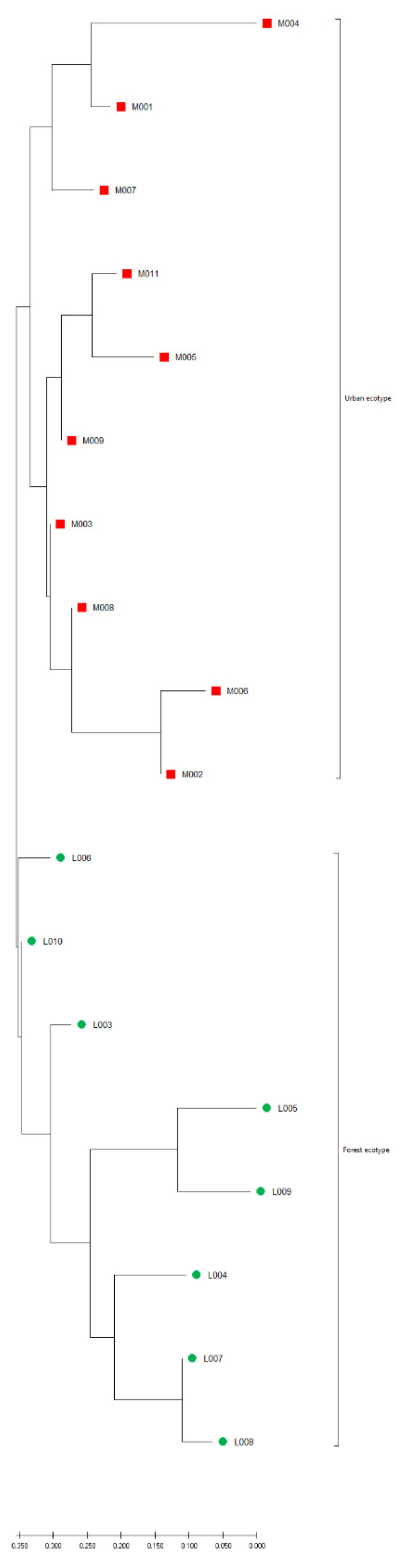
Nei’s distance relationships among nests and ecotypes. The distance was inferred using the Neighbor-Joining method. The optimal tree with a sum of branch length of 157,714,544 is shown. The tree is drawn to scale with branch lengths having the same units as those of the Nei’s distances used to infer the distance matrix. U—Urban nest, L—Forest nest; the numbers shown the different nests.

The division into two groups is even more visible on the genetic distance tree （Figure 7). The similarity of birds within a given ecotype varied, but in general, it can be seen that birds belonging to the urban ecotype differed from individuals belonging to the forest ecotype.

## 4. Discussions

### 4.1. Population Variability

Compared to the Scandinavian population of peregrine falcons that was studied by Nesje et al. [17], who analyzed 24 unrelated individuals and 64 dyads of full-siblings and potential full-siblings, 9 out of 10 markers had a greater number of alleles, and in the case of marker NVHfp79_4, the number of alleles was equal in both studies. For 8 out of 10 markers observed, heterozygosity was greater in the Scandinavian population.

In another study, three groups of Scandinavian birds were analyzed [19]: historical (*n* = 38), captive (*n* = 20), and contemporary (*n* = 44) wild populations. Compared to the historical populations originating from Denmark and Norway, for 6 out of 10 markers, the number of alleles was greater in our study, and for two markers, the number of alleles was lower. The observed heterozygosity was greater in the historical Scandinavian population for seven markers. Compared with captive and contemporary populations, the number of alleles in our population was greater for seven markers in both these comparisons. The observed heterozygosity was lower for seven markers; however, only one marker had a greater observed heterozygosity in both captive and contemporary populations.

Similarly, as with the Czech falcon population [1], the Polish wild population was not found to differ visibly from the breeding one. Individuals from both Polish populations were classified into both populations estimated in STRUCTURE. The reason for this can be found in the Polish reintroduction plan, which is based on individuals originating from all breeding centers included in our analysis and on cooperation between European breeders, resulting in a flow of birds between breeding centers [7].

The microsatellite analysis revealed a low level of genetic variation in the Fiji falcon population associated with a lack of gene flow from populations inhabiting the nearest islands and leading to differences among the analyzed populations [36]. Microsatellites are proving to be capable of distinguishing between populations. In our study, gene flow between the analyzed populations was confirmed in the cases of both breeder-breeder and breeder-environment flow. It is also likely that gene flow will appear between populations living in neighboring countries, such as between Germany and the Czech Republic or the Polish population. However, such a flow may prove to be difficult to detect due to the use of birds originating from breeding centers that also provide birds for the Polish reintroduction program in these countries. Further analyses using more genetic markers and data from ornithological rings are needed.

A low level of heterozygosity was observed across all analyzed samples in loci NVHfp82_2, NVHfp79_4 and NVHfp5 (Table 1) in our study. Despite the low level of genetic variation among previously analyzed peregrine falcon populations shown in other studies [1,17,19], the level of heterozygosity in the discussed loci was greater in all other populations, excepted for the Fijian population, although Fijian population was monomorphic [36]. In further studies, the rejection of these markers should be carefully considered. The majority of the studied group of birds (262 individuals) were captive breeding animals. The proximity of breeding centers and the exchange of birds may reduce the heterozygosity of species whose genetic variation shows a poor population structure (i.e., differentiation) consistent with incomplete sorting among rapidly evolving lineages [37].

### 4.2. Bird Release Point

The main aim of the Polish reintroduction program has been to restore the tree-nesting population. Our wild population analysis shows genetic differences between ecotypes. The genetic distance tree shows individuals representing both ecotypes. Their diversity is mainly due to isolation—birds from the urban ecotype do not migrate to forests. However, the reintroduction process may change this relationship. Birds introduced in urban areas show greater diversity than tree nesting birds. However, no correlation between the genetic distance and the distance of the studied nests was found, which clearly indicates that in most cases there is no natural gene flow. An analysis performed on the Polish population of the Eurasian coot (*Fulica atra*) showed that long-established urban populations differ significantly from the forest population, although no significant differences between newly established urban populations and the forest population were identified [38]. The differences between the studied populations of wild falcons may become blurred as a result of their ongoing reintroduction.

### 4.3. Stock of Falcons for Reintroduction

Due to the lack of wild falcons, breeding materials for Polish falcon breeding centers were brought from Western European breeding sources [16]. In the STRUCTURE analysis, individuals were classified into two groups independently of the country of origin when wild birds were included. When wild birds were excluded, individuals were classified into two groups, and in both cases, classification seemed to be independent from the country of breeding. European breeders cooperate with each other [7]. Bird exchange between breeders explains why we were only able to distinguish between two genetic groups of captive falcons (despite the fact that the analyzed individuals came from 47 breeding sites located in five countries).

### 4.4. Implications for Conservation

Genetic monitoring of populations can lead to improved population management (see [39]). Some data suggest that decades of breeding in captivity has had an impact on the falcon genome due to the creation of selection regimes, even though this was probably not a deliberate act [40]. Selective breeding to obtain certain traits (such as size or pursuit patterns) desired by falconers is likely to have had an impact on the falcon genome; moreover, captive falcons have sometimes been hybridized [41]. It is crucial to monitor captive individuals to be used for reintroduction as well as the wild population. Our analysis may be the first step toward improving the management of the Polish population of peregrine falcons. Estimation of the population’s genetic variability and number of groups provides insight into the effects of a reintroduction plan as well as basic knowledge that enables the use of more powerful (and expensive) genetic tools such as DNA sequencing or microarray analyses. Equipped with this knowledge, study groups can be optimally designed for these analyses. We are cooperating with the Society for Wild Animals “Falcon”, which is a non-governmental organization that has been responsible for peregrine falcon restitution in Poland since 2010. On the one hand, this provides wide access to biological samples, and on the other, it provides the opportunity to apply the results of our population studies.

## Figures and Tables

**Table 1:** Analysis of genetic variability.

Locus	Allele Ranges	*n*	Na	Ne	Ho	He	F_ST_
NVHfp107	114–208	350	6	1.950	0.454	0.487	0.042
NVHfp13	93–103	350	9	4.714	0.554	0.788	0.030
NVHfp46_1	117–122	350	6	2.925	0.540	0.658	0.055
NVHfp5	104–108	352	3	1.489	0.057	0.328	0.159
NVHfp54	104–116	348	7	2.395	0.382	0.528	0.049
NVHfp79_4	104–142	352	3	1.982	0.000	0.495	0.029
NVHfp82_2	134–140	352	4	1.143	0.085	0.125	0.060
NVHfp86_2	140–145	343	5	2.146	0.370	0.534	0.045
NVHfp89	116–132	347	9	4.467	0.617	0.776	0.052
NVHfp92_1	110–130	349	9	2.617	0.298	0.618	0.075

*n*—number of scored individuals, Na—observed number of alleles, Ne—effective number of alleles, Ho—observed heterozygosity, He—expected heterozygosity, F_ST_—F-statistic.

## Data Availability

The data presented in this study are available on request from the corresponding author.

## References

[1] Bryndová M., Putnová L., Bartoňová P., Kaplanová K., Urban T. (2012). Genetic differences between wild and captive populations of the peregrine falcon (*Falco peregrinus*) and the saker falcon (*Falco cherrung*) living in the Czech Republic. J. Agric. Sci. Technol. B.

[2] Wegner P., Kleinstäuber G., Baum F., Schilling F. (2005). Long-term investigation of the degree of exposure of German peregrine falcons (*Falco peregrinus*) to damaging chemicals from the environment. J. Ornithol..

[3] Brown J.W., Van Coeverden De Groot P.J., Birt T.P., Seutin G., Boag P.T., Friesen V.L. (2007). Appraisal of the consequences of the DDT-induced bottleneck on the level and geographic distribution of neutral genetic variation in Canadian peregrine falcons, *Falco peregrinus*. Mol. Ecol..

[4] Crick H.Q.P., Ratcliffe D.A. (1995). The peregrine *Falco peregrinus* breeding population of the United Kingdom in 1991. Bird Study.

[5] Olsen P., Emison B., Mooney N., Brothers N. (1992). DDT and dieldrin: Effects on resident peregrine falcon populations in south-eastern Australia. Ecotoxicology.

[6] Mizera T., Sielicki J. (1995). The peregrine falcon *Falco peregrinus* in Poland—Its situation and perspectives for reinstatment. Acta Ornithol..

[7] Sielicki S., Sielicki J., Sielicki J., Mizera B. (2009). Restoration of peregrine falcon in Poland 1989–2007. Peregrine Falcon Populations—Status and Perspectives in 21th Century.

[8] Cichocki W. (1986). Some species of breeding birds in Tatra National Park. Parki Narodowe i Rezerwaty Przyrody.

[9] Ivanovsky V., Vintchevski A., Sielicki J., Mizera B. (2009). Status of the peregrine falcon in Belarus. Peregrine Falcon Populations—Status and Perspectives in 21th Century.

[10] Brambilla M., Rubolini D., Guidali F. (2006). Factors affecting breeding habitat selection in a cliff-nesting peregrine *Falco peregrinus* population. J. Ornithol..

[11] Kirmse W., Chancellor R.D., Meyburg B.U. (2004). Tree-nesting Peregrines *Falco p. peregrinus* in Europe did not recover. Raptors Worldwide.

[12] Sielicki S., Sielicki J., Szymak U., Sianko P. (2016). Falconry and the restoration of the peregrine falcon in Poland in 1990–2015. Falconary—Its Influence on Biodiversity and Cultural Heritage.

[13] Ławicki Ł., Sielicki S. (2019). Restoration of the tree-nesting population of the peregrine falcon *Falco peregrinus* in Pomerania. Ornis Pol..

[14] Banks A.N., Crick H.Q.P., Coombes R., Benn S., Ratcliffe D.A., Humphreys E.M. (2010). The breeding status of peregrine falcons *Falco peregrinus* in the UK and Isle of Man in 2002. Bird Study.

[15] Verdejo J., López-López P. (2008). Long-term monitoring of a peregrine falcon population: Size, breeding performance and nest-site characteristics. Ardeola.

[16] Wisniewski G. (1995). Programme for the reinstatement of the peregrine falcon *Falco peregrinus* in Poland. Acta Ornithol..

[17] Nesje M., Røed K.H., Bell D.A., Lindberg P., Lifjeld J.T. (2000). Microsatellite analysis of population structure and genetic variability in peregrine falcons (*Falco peregrinus*). Anim. Conserv..

[18] Nittinger F., Gamauf A., Pinsker W., Wink M., Haring E. (2007). Phylogeography and population structure of the saker falcon (*Falco cherrug*) and the influence of hybridization: Mitochondrial and microsatellite data. Mol. Ecol..

[19] Jacobsen F., Nesje M., Bachmann L., Lifjeld J.T. (2008). Significant genetic admixture after reintroduction of peregrine falcon (*Falco peregrinus*) in Southern Scandinavia. Conserv. Genet..

[20] Bell D.A., Griffiths C.S., Caballero I.C., Hartley R.R., Lawson R.H. (2014). Genetic Evidence for Global Dispersal in the peregrine falcon (*Falco peregrinus*) and Affinity with the taita falcon (*Falco fasciinucha*). J. Raptor Res..

[21] Rutkowski R., Rejt Ł., Tereba A., Gryczyńska-Siemiatkowska A., Janic B. (2010). Population genetic structure of the European kestrel *Falco tinnunculus* in Central Poland. Eur. J. Wildl. Res..

[22] Groombridge J.J., Dawson D.A., Burke T., Prys-Jones R., Brooke M., de L. Brooke M., Shah N. (2009). Evaluating the demographic history of the Seychelles kestrel (*Falco araea*): Genetic evidence for recovery from a population bottleneck following minimal conservation management. Biol. Conserv..

[23] Peakall R., Smouse P.E. (2006). GenAlEx 6: Genetic analysis in Excel. Population genetic software for teaching and research. Mol. Ecol. Notes.

[24] Peakall R., Smouse P.E. (2012). GenAlEx 6.5: Genetic analysis in Excel. Population genetic software for teaching and research-an update. Bioinformatics.

[25] Marshall T.C., Slate J., Kruuk L., Pemberton J.M. (1998). Statistical confidence for likelihood-based paternity inference in natural populations. Mol. Ecol..

[26] Nei M. (1978). Estimation of average heterozygosity and genetic distance from a small number of individuals. Genetics.

[27] Sneath P.H.A., Sokal R.R. (1973). Numerical Taxonomy.

[28] Saitou N., Nei M. (1987). The neighbor-joining method: A new method for reconstructing phylogenetic trees. Mol. Biol. Evol..

[29] Kumar S., Stecher G., Li M., Knyaz C., Tamura K. (2018). MEGA X: Molecular Evolutionary Genetics Analysis across computing platforms. Mol. Biol. Evol..

[30] Falush D., Stephens M., Pritchard J.K. (2007). Inference of population structure using multilocus genotype data: Dominant markers and null alleles. Mol. Ecol. Notes.

[31] Hubisz M.J., Falush D., Stephens M., Pritchard J.K. (2009). Inferring weak population structure with the assistance of sample group information. Mol. Ecol. Resour..

[32] Pritchard J.K., Stephens M., Donnelly P. (2000). Inference of population structure using multilocus genotype data. Genetics.

[33] Falush D., Stephens M., Pritchard J.K. (2003). Inference of population structure using multilocus genotype data: Linked Loci and Correlated Allele Frequencies. Genetics.

[34] Earl D.A., vonHoldt B.M. (2012). STRUCTURE HARVESTER: A website and program for visualizing STRUCTURE output and implementing the Evanno method. Conserv. Genet. Resour..

[35] Evanno G., Regnaut S., Goudet J. (2005). Detecting the number of clusters of individuals using the software STRUCTURE: A simulation study. Mol. Ecol..

[36] Talbot S.L., Palmer A.G., Sage G.K., Sonsthagen S.A., Swem T., Brimm D.J., White C.M. (2011). Lack of genetic polymorphism among peregrine falcons *Falco peregrinus* of Fiji. J. Avian Biol..

[37] White C.M., Cade T.J., Enderson J.H. (2013). Peregrine Falcons of the World.

[38] Minias P., Włodarczyk R., Minias A., Dziadek J. (2017). How birds colonize cities: Genetic evidence from a common waterbird, the Eurasian coot. J. Avian Biol..

[39] Johnson J.A., Talbot S.L., Sage G.K., Burnham K.K., Brown J.W., Maechtle T.L., Seegar W.S., Yates M.A., Anderson B., Mindell D.P. (2010). The use of genetics for the management of a recovering population: Temporal assessment of migratory peregrine falcons in North America. PLoS ONE.

[40] Wilcox J.J.S., Boissinot S., Idaghdour Y. (2019). Falcon genomics in the context of conservation, speciation, and human culture. Ecol. Evol..

[41] Fleming L.V., Douse A.F., Williams N.P. (2011). Captive breeding of peregrine and other falcons in Great Britain and implications for conservation of wild populations. Endanger. Species Res..

